# Perimenstrual asthma: from pathophysiology to treatment strategies

**DOI:** 10.1186/s40248-016-0065-0

**Published:** 2016-08-01

**Authors:** Alessandra Graziottin, Audrey Serafini

**Affiliations:** 1 Center of Gynecology and Medical Sexology, San Raffaele Resnati Hospital, Milan, Italy; 2 Gynecologist, Milan, Italy; 3 Via Enrico Panzacchi 6, 20123 Milan, Italy

**Keywords:** Menstrual asthma, Mastcells, Estrogens, Inflammation, Oral contraception, Hormone free interval

## Abstract

The prevalence of asthma is about 9,7 % in women and 5,5 % in men.

Asthma can deteriorate during the perimenstrual period, a phenomenon known as perimenstrual asthma (PMA), which represents a unique, highly symptomatic asthma phenotype. It is distinguished from traditional allergic asthma by aspirin sensitivity, less atopy, and lower lung capacity.

PMA incidence is reported to vary between 19 and 40 % of asthmatic women. The presence of PMA has been related to increases in asthma-related emergency department visits, hospitalizations and emergency treatment including intubations.

It is hypothesized that hormonal status may influence asthma in women, focusing on the role of sex hormones, and specifically on the impact of estrogens’ fluctuations at ovulation and before periods.

This paper will focus on the pathophysiology of hormone triggered cycle related inflammatory/allergic events and their relation with asthma. We reviewed the scientific literature on Pubmed database for studies on PMA. Key word were PMA, mastcells, estrogens, inflammation, oral contraception, hormonal replacement therapy (HRT), and hormone free interval (HFI).

Special attention will be devoted to the possibility of reducing the perimenstrual worsening of asthma and associated symptoms by reducing estrogens fluctuations, with appropriate hormonal contraception and reduced HFI. This novel therapeutical approach will be finally discussed.

## Background

Asthma is one of the most common chronic respiratory diseases. According to the Centers for Disease Control and Prevention (CDC) and to 2000 Behavioral Risk Factor Surveillance System, the prevalence of asthma ranges from 9.1 to 9,7 % in adult women and 5.1 to 5,5 % in men, i.e. women present almost twice the risk men have [1, 2]. The increased vulnerability to inflammatory and autoimmune disease women experience after puberty is considered secondary to the different effect sex hormones have on the inflammatory defense system, and specifically on mast-cells (MC) [3, 4] and eosinophils cells. Fluctuations of estrogens trigger the mast-cell degranulation, whilst testosterone and other androgens are credited to have a stabilizing effect. Perimenstrual fluctuations of sex hormones in women are considered responsible for the specific worsening of many different perimenstrual symptoms and of specific inflammatory [5], autoimmune [6, 7] and pain related conditions, such as headache and pelvic pain [8–11].

The focus of this paper will be twofold: 1. To analyze the pathophysiology of menstrual cycle related events (ovulations and periods) and their relation with ovulatory and, with specific focus, PMA; 2. To discuss preventive strategies aiming at reducing the cycle – related inflammatory/allergic worsening of asthma and associated symptoms by reducing estrogens fluctuations, with appropriate hormonal contraception and reduced HFI.

## Review

### Asthma in women’s lifespan: windows of vulnerability

- ➢ Puberty: asthma is more common and severe in women during and after puberty. Prevalence is higher in women with early menarche [12]
- ➢ Ovulation: asthma exacerbations begin more often during the preovulatory (28 %) period in women with this chronic condition. Ovulation associated sex hormones’ fluctuations may trigger asthmatic crisis in vulnerable women [12–15].
- ➢ Menstruation: asthma can deteriorate during the perimenstrual period, a phenomenon known as perimenstrual asthma (PMA) which is usually much more severe and troublesome than the periovulatory worsening [16].
- ➢ Pregnancy: the clinical course of asthma during pregnancy is variable: it may worsen in about one-third of pregnant women, and may improve in about one-quarter [17–20]. It appears that mild asthma is likely to improve, whereas more severe forms of the disease frequently worsen [17–20]. Asthma generally improves during the last 4 weeks of gestation, and attacks are very infrequent during labor and delivery [17, 20]
- ➢ Puerperium: changes in the asthmatic state during pregnancy generally last up to 3 months after delivery [17, 20].
- ➢ Menopause: postmenopausal women present a significantly lower risk of developing asthma than premenopausal women of similar age [17, 21]
- ➢ HRT: results in the effect of HRT on asthma are controversial. In one study, ever using HRT was associated with increased risk of hospital admission for asthma. This risk was highest with the longest duration of HRT use. The risk was significantly increased for all types of HRT regimens [22]

In other studies neither the discontinuation nor reinitiation of HRT in the postmenopausal period has any effect on airway obstruction in asthmatic women [21, 23].

### Definition of PMA

A clear definition of PMA does not exist in the literature. Murpyh et al. investigated the following types of PMA definitions: self-reported PMA, increased symptoms, increased medication use, decreased peak flow or a combination of changes in symptoms, medication and peak flow. The variability of the definition of PMA affects the recollection of the data in the different studies and affects the prevalence in different populations [24].

Generally, the deterioration of asthma during the luteal phase and/or during the first days of menstruation, is characterized by deterioration of lung function tests [25].

PMA represents a unique, highly symptomatic, and exacerbation-prone asthma phenotype associated with aspirin sensitivity, less atopy (less IgE level), and lower forced vital capacity, that distinguish it from traditional allergic asthma [26].

### Prevalence of PMA

PMA incidence is reported to be as high as 40 % of asthmatic women [27].

In population based studies, rates of admissions to hospital for asthma are similar by sex in the early teenage years [13, 28, 29] but three times higher in women than in men aged 20–50 years. After menopause the incidence of asthma falls and equalizes again with men [13, 28, 29].

The presence of PMA has been related to increases in asthma-related Emergency Department (ED) visits, hospitalizations, intensive care unit (ICU) admissions, intubations, and near-fatal and fatal events [13, 26].

ED visits occur more commonly among women in the preovulatory (28 %) and perimenstrual (27 %) phases (p = 0.004) [13].

These findings, together with the growing body of evidence for sex differences in asthma [12, 13], support the hypothesis that hormonal status may influence asthma in women, focusing on the role of sex hormones, and specifically on the impact of estrogens’ fluctuations at ovulation and before periods [13].

### Why do ovulation and menstruation trigger asthma attacks?

New data suggest that specific pathophysiologic mechanisms could be responsible for PMA attacks. An updated physiology of the cycle related events will be briefly reviewed here, to set the scenario which leads to PMA. It focuses on the inflammatory nature of the menstrual process, currently understood as the “genital sign of systemic endocrine and inflammatory events” [3, 4].

- ➢ The updated physiology of estrogen’ fluctuations at ovulation and menstruation

The physiology of the menstrual cycle is characterized by fluctuating levels of luteinizing hormone (LH), follicle-stimulating hormone (FSH), oestradiol, progesterone and testosterone. The perimenstrual phase is characterized by a decline in progesterone and oestradiol levels [30], which triggers MC degranulation at the basal layer of the endometrium. This induces a local (with endometrial tissue breakdown and menstruation) and a *systemic* inflammation (with MC and eosinophil degranulation and consequent increase in inflammatory markers in tissues where hyperactive MC are already present, such as the lung/bronchial tissues of an asthmatic woman) [3].

- ➢ Effect of estrogens on immune cells

Sex hormones are effective modulators of immune and inflammatory responses [31]. Estrogens significantly influence the incidence and/or the course of several autoimmune diseases, as well as bacterial and parasitic infectious processes [31–34]. They exert their actions through the estrogen receptor (ER)-alpha and -beta [31, 34–36], which are expressed by several immune cells [37].

- Eosinophils : Chronic administration of E2 to ovariectomized mice prevents eosinophilia in sterile peritonitis induced by thyoclicolate challenge (with a sixfold decrease in circulating eosinophils as compared with untreated ovariectomized mice) [31].
- MC: MC are considered to be the chief protagonists in the clinical scenario of inflammation and pain [3, 4, 38–40]. MC are present in the endometrium and myometrium and are predominantly localized to the basal layer [41]. MC are upregulated in response to a wide range of stimuli, including neurogenic factors, fluctuating oestrogen levels and menstrual blood in the tissue [9]. Once activated, MC degranulate and release a range of inflammatory mediators which perpetuate the immune response [9]. Sex hormones regulate MC functionality and distribution in several tissues [42–47], both in physiological and pathological conditions. In this regard, a relationship between female sex hormones, MC and development of asthma and allergy has been suggested [46–49]. Furthermore, the presence of sex steroid receptors on MC indicates that sex hormones may exert their biological effects by binding to these receptors [49].

### Asthma and respiratory atopy

Asthma, allergies and other manifestations of atopy have also been shown to fluctuate throughout the menstrual cycle [50].

The female immune response changes throughout the menstrual cycle. Many studies support this theory:

- ➢ One study examining skin prick testing in women with aero-allergens reported significantly increased wheel-and- flare responses on days 12–16 of the menstrual cycle which correspond to ovulatory peak estrogen levels [51].
- ➢ The menstrual phase has also been shown to influence nasal reactivity, as the period of peak estrogen is correlated with the nasal mucosa becoming hyperreactive to histamine [52].
- ➢ Bronchial hyperreactivity is more likely in the perimenstrual period than at other points in the cycle; PMA seems to be closely linked to total IgE levels but not to specific allergens [53].
- ➢ Atopic asthma, a specific asthma phenotype, is often associated to an irregular menstrual cycle [54, 55]

### Asthma and combined contraceptive pill

Studies have demonstrated a role for both exogenous estrogen and progesterone in the management of PMA [25, 56–58]. In a prospective study on postpubertal women with asthma, the 106 women using oral contraception (OC) had reduced asthma symptoms, improved pulmonary function, and improved asthma control compared with those not using OC [57].

A study from Tan et al. compared airway reactivity to adenosine monophosphate (AMP) in female asthmatics with natural menstrual cycles and those taking the OC (with 21 days of active treatment followed by a seven-day break). There was a significant increase in airway reactivity in the group with natural menstrual cycles in the luteal phase, coincident with the increase in progesterone and estradiol. In the OC group the hormonal profile was stable, and so was bronchial reactivity. They concluded that asthmatic patients receiving the OC had attenuated cyclical change in airway reactivity as well as reduced diurnal peak expiratory flow rate variability, which was associated with suppression of the normal luteal phase rise in sex-hormones [58].

### How and why can OC reduce PMA

How this beneficial effect is obtained? One possible explanation could be the regulation of the immune system. An exaggerated Th2-driven response is believed to lead the inflammatory allergic reaction of asthma. Particularly, Th2 cytokines induce the production of IgE and mast cells, activation and recruitment of eosinophils, and airway hyperreactivity and mucus secretion [59]. Of major importance are induced regulatory T cells (iTregs), which can inhibit or suppress the effector function of T helper cells and their tolerance to environmental allergens [59–61] thus preventing the induction or reducing the severity of asthma [59–61].

Sex hormones may directly contribute to the perturbed development of iTregs perpetuating the asthmatic inflammation in an at-risk female population. Consistent with this possibility, the lower and more stable levels of circulating hormones in women with asthma using OC were associated with higher percentages of iTregs when compared with non-OC patients [59–61].

These results identify an impact of sex hormones in the capacity of T cells to polarize towards a regulatory phenotype and suggest the regulation of peripheral T cell lineage plasticity as a potential mechanism underlying the beneficial effects of OC in women with asthma [59–61].

### Preventive strategies for PMA

Understanding that the menstrual fall of estradiol and progesterone triggers asthmatic crises in vulnerable women may suggest new preventive strategies which are pathophysiologically oriented, such as stabilizing estradiol and progesterone/progestins levels and reducing the hormone free interval (HFI), when OC is considered. Prospective controlled studies are needed to test this working hypothesis.

Menstrual symptoms are complained of as well, although with reduced intensity, during the HFI. The original interval of seven days has been progressively reduced to 4 and to 2, with a significant reduction of menstrual symptoms, or deleted for three or more months (“extended regimens”, when the woman may have periods every 91 days). A solid evidence (Harmony I and II) [62, 63] supports the significant advantages in terms of menstrual headache and chronic pelvic pain improvement by reducing HFI.

### Advantages of providing stable hormone levels in place of monthly hormonal fluctuations

Menstrual bleeding is not biologically necessary in women taking OC. Rather, for physical and mental good health, women require appropriate, stable levels of oestrogens, progesterone and androgens [3, 4].

OC have traditionally been administered with 21 days of active treatment followed by a seven-day break (a ‘21/7’ pattern) during which withdrawal bleeding occurs. It is worth noting that the 7 days HFI, designed in the pioneering years of OC, had a psychosocial, not a medical reason. It was simply needed to reassure women that they were not pregnant, amenorrhea being for millennia the first symptom and sign of pregnancy. Today this is no more the case. Data [64] show a strong cultural effect: women in high income country tend to have a very flexible relationship with their periods, the Netherland leading the ranking with 85 % asking to postpone/avoid the cycle, vs 10 % of women in Turkey. Newer formulations are available in which the HFI is reduced or in some cases eliminated altogether.

There are three major rationales for a shorter HFI:

1. To reduce menstrual symptoms
  Shortening HFI reduces genital and systemic inflammation and symptoms associated with hormone withdrawal. In a study of nearly 300 women, pelvic pain, headache, breast tenderness, bloating/swelling and analgesic use were all significantly more frequent during the seven-day HFI than in the 21 days of active treatment [65]. OC with a shortened HFI provides markedly greater suppression of ovarian activity and limits the extent of exogenous hormonal fluctuations throughout the cycle, and has been shown to reduce or eliminate these symptoms [65].
2. To provide more powerful ovarian suppression and consequently increase contraceptive efficacy [65].
3. To reduce heavy menstrual bleeding (HMB)

HMB affects around one in three women and has a significant negative impact on quality of life [66, 67]. The pill with oestradiol valerate and dienogest (E_2_V/DNG), administered on a 26/2 regimen, significantly reduces the duration and severity of the menstrual bleeding and it is the only one approved for the treatment of HMB and consequent anemia.

### Anemia is a risk factor for asthma

In one study from Ramakrishnan et al., anemic children resulted 5.75 times more susceptible to asthmatic attacks when compared with non-anemic children [68].

Moreover, anemia can worsen dyspnea related to asthma, severely impairing oxygen delivery because the bulk of oxygen carried in the blood is hemoglobin-bound [69].

An adequate level of iron has a protective effect on the respiratory system. A study from Hale et al., proved that mice fed an iron-supplemented diet had markedly decreased allergen-induced airway hyperreactivity, eosinophil infiltration, and production of pro-inflammatory cytokines, compared with control mice on an unsupplemented diet that generated mild iron deficiency but not anemia. In vitro, iron supplementation decreased MC granule content, IgE-triggered degranulation, and production of pro-inflammatory cytokines post-degranulation. Iron supplementation can decrease the severity of allergic inflammation in the lung, via multiple mechanisms that affect MC activity [70].

### Evidence supporting the benefits of reducing the HFI

#### From 7 to 4 days

Shortening of the 7-day HFI (21/7) from 7 to 4 days (24/4): all of the 24/4 regimens, including EE/DSPR, EE/Noretindrone acetate and E2/NOMAC, have shown comparable or more favourable effects than 21/7 regimens in terms of efficacy and safety profiles.

This translates into improved menstrual symptoms control: significant reduction of PMS symptoms [71], dysmenorrhoea [71], premenstrual dysphoria disorder (PMDD) [72], and improvements in quality of life [4].

### From 7 to 2 days

The benefits of shortening the HFI may be even greater when further reducing it from 7 to 2 days. E_2_V/DNG is a 26/2-pattern COC that features quadriphasic dosing, delivering a reducing dose of oestrogen over days 1–26 and an increasing dose of progestogen over days 3–24, followed by two hormone-free days. E_2_V/DNG is the first OC approved for the treatment of HMB due to endometrial dysfunction. Benefits include:

- ➢ Contraceptive efficacy [73]
- ➢ Reduction of HMB, thus reducing anemia that potentiate asthma with an independent mechanism [68–70, 74, 75].

### Role of the E_2_V/DNG pill in reducing menstrual symptoms

E_2_V/DNG reduces the intensity of menstrual symptoms by offering constant levels of oestradiol around 50 picograms/mL (range 37–62), thus modulating and reducing the MC degranulation triggered by a more dramatic hormonal fall typical of the natural cycle (when oestradiol moves from 50 to 100 pg/mL after periods, up to 400–500 pg/mL at ovulation and around 200 pg/mL in the luteal phase) [4].

### Depression associated with HMB

Reducing the blood loss, E2V/DNG decreases iron-loss anemia, which may be a risk factor for anxiety [76, 77], poor concentration, attention and memory [78], fatigue [79].

The effect of E_2_V/DNG on maintaining iron levels has a positive effect on the dopaminergic system, which is involved in ameliorating mood, assertiveness, physical and mental energy, and reducing the risk of depression. Moreover, it can reduce the periodical asthmatic crisis, which may be so important to cause a post-traumatic stress disorder [80], and the need of corticosteroids boluses and chronic therapy thus reducing the collateral effects such as weight augmentation [81]

### Menstrual migraine and pelvic pain

E_2_V/DNG has shown a reduction in systemic symptoms associated with the HFI such as headache [82].

## Conclusions

Asthma is one of the most common chronic respiratory diseases. The prevalence of asthma ranges from 9.1 to 9,7 % in adult women and 5.1 to 5,5 % in men [1, 2].

PMA is usually described as cyclical deterioration of asthma during the luteal phase and/or during the first days of menstruation [25, 26], and is reported to be about 19 % of asthmatic women, while other studies reported the incidence to be as high as 40 % [27].

These menstrual phase findings support the hypothesis that hormonal status may influence asthma in women, focusing on the role of sex hormones, and specifically on the impact of estrogens’ fluctuations at ovulation and before periods [28].

Women today have many more periods in their lifetime than their ancestors. Menstrual bleeding is not biologically necessary in women taking hormonal contraceptives. Furthermore, it may be advantageous for the women to have more stable levels of hormones throughout the cycle. The monthly fluctuations in oestrogens, progesterone and androgens are associated with a range of symptoms, both genital (i.e. vaginal bleeding, heavy menstrual bleeding, dysmenorrhoea and pelvic pain) and systemic (depression, fatigue, headache, IBS symptoms, asthma and allergy), triggered by a local and systemic rise in inflammatory molecules released by MC when estrogens drop. These symptoms arise through a complex interaction between the endocrine and immune systems.

OCs traditionally feature a 7-day HFI, during which menstruation occurs. Formulations with a shorter HFI have recently been developed with the aim of offering a reduction in hormone withdrawal-associated symptoms together with more powerful ovarian suppression. E_2_V/DNG is administered on a 26/2 regimen and has been shown to offer high contraceptive efficacy together with a reduction in heavy menstrual bleeding, improvement in hormone withdrawal-associated symptoms and improvement in sexual function. E_2_V/DNG may, therefore, be a good alternative to conventional 21/7 COCs for women with bothersome COC- or menstruation-related symptoms, as exacerbation of asthma crisis.

Prospective controlled studies are needed to confirm the working hypothesis, supported by preliminary evidence, that reduction of HFI translates into a significant reduction of PMA; to evaluate in head to head studies if a regimen with natural estradiol-valerate/dienogest with 2 days interval has a different impact on PMA in comparison to a continuous regimen with EE 30 mcg and levonorgestrel.
